# Life-Threatening Intrapulmonary Hemorrhage due to Vancomycin-Induced Thrombocytopenia: A Case Report

**DOI:** 10.1155/2020/8890335

**Published:** 2020-09-30

**Authors:** Haneen Abdalhadi, Yazan Fahmawi, Abhijin Das, Brian Fouty

**Affiliations:** ^1^Department of Internal Medicine, University of South Alabama, Mobile, AL, USA; ^2^Division of Nephrology, University of South Alabama, Mobile, AL, USA; ^3^Division of Pulmonary and Critical Care, University of South Alabama, Mobile, AL, USA

## Abstract

Thrombocytopenia is a rare and sometimes life-threatening complication of Vancomycin. A 52-year-old male patient with acute kidney injury was treated with Vancomycin for ventilator-associated pneumonia. Three days later, his platelets decreased from 172 × 10^9^/L to 3 × 10^9^/L over a 36-hour period. The patient developed significant intrapulmonary bleeding leading to profound hypoxemia. Workup was negative for thrombotic thrombocytopenic purpura, disseminated intravascular coagulopathy, atypical hemolytic uremic syndrome, heparin-induced thrombocytopenia, and autoimmune diseases. All recently started medications were discontinued, and the patient was started empirically on methylprednisolone and intravenous immunoglobulin. The patient's platelets increased, and his airway bleeding stopped within 48 hours; his platelet count returned to normal by 18 days. Vancomycin-dependent anti-platelet antibodies were identified in the patient's serum by flow cytometry. Thrombocytopenia is an underrecognized complication of Vancomycin that can lead to life-threating bleeding. Stopping Vancomycin may be sufficient to reverse the thrombocytopenia in patients with normal renal function, but more aggressive measures such as steroids, IVIG, and dialysis may be required to stop bleeding and reverse thrombocytopenia in patients with underlying kidney injury who cannot effectively excrete Vancomycin.

## 1. Introduction

Vancomycin is a glycopeptide antibiotic commonly used to treat severe gram-positive bacterial infections, especially those caused by methicillin-resistant *Staphylococcus aureus* and coagulase-negative *Staphylococci* [1]. Nephrotoxicity, ototoxicity, red-man syndrome, and reversible neutropenia are well-known side effects of Vancomycin [2–4]. Thrombocytopenia is a less commonly described complication of Vancomycin that is frequently overlooked by physicians likely due to the presence of other potential causes of thrombocytopenia in critically ill patients, such as sepsis and concomitant heparin use. The lack of an easily available point-of-care assay makes it a difficult diagnosis to confirm. Herein, we present a case of severe symptomatic Vancomycin-induced thrombocytopenia (VIT) that was confirmed by identifying Vancomycin-dependent anti-platelet antibodies in the patient's serum.

## 2. Case Presentation

**Figure 1:**
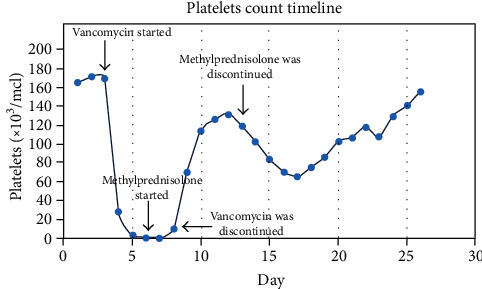
The platelets trend during patient hospitalization.

A 52-year-old Caucasian male with diabetes mellitus, hypertension, and coronary artery disease was initially admitted to the Intensive Care Unit for pulmonary edema secondary to hypertensive emergency with a blood pressure of 254/157 mmHg. He required intubation for acute hypoxemic respiratory failure. The patient developed acute kidney failure (AKI) in the hospital. Workup for his AKI, including renal ultrasound and serologies, failed to identify a specific cause for his AKI. There was no evidence of vasculitis clinically or on renal biopsy. He was begun on hemodialysis. During this entire period, his platelet count was normal. He developed a ventilator-associated pneumonia and was begun on empiric Vancomycin and Meropenem; no pathogen was ever identified in the patient's sputum or blood. Three days after starting these antibiotics, his platelets decreased precipitously from 172 × 10^9^/L to 3 × 10^9^/L over a 36-hour period (Figure 1). No schistocytes were seen on the peripheral smear, and there was no clinical evidence of hemolysis. Additional studies to evaluate for thrombotic thrombocytopenic purpura, disseminated intravascular coagulopathy, atypical hemolytic uremic syndrome, heparin-induced thrombocytopenia, and autoimmune diseases were performed; all came back negative. The patient's platelet count decreased to 1 × 10^3^/*μ*L despite multiple platelet transfusions. The patient developed an intrapulmonary hemorrhage and became severely hypoxemic despite receiving 100% oxygen. A presumptive diagnosis of drug-induced immune thrombocytopenia was made. All medications that had been recently started, including Vancomycin and Meropenem, were discontinued. Due to his life-threatening airway bleeding, the patient was treated with high-dose methylprednisolone (500 mg/day) and a five-day course of intravenous immunoglobulin (IVIG). His platelet count began to increase 48 hours after steroids were begun, and his intrapulmonary bleeding resolved with improvement in his oxygenation. After 7 days, his platelet count was 160 × 10^9^/L. The platelet count decreased to less than 100 × 10^9^/L after methylprednisolone was stopped. Therefore, it was restarted for an additional 10 days. Incubation of the patient's serum with platelets from normal donors resulted in the binding of IgG and IgM antibodies in the presence of Vancomycin, but not in its absence, supporting a diagnosis of Vancomycin-induced thrombocytopenia (VIT).

## 3. Discussion

Thrombocytopenia is an underrecognized complication of Vancomycin that can lead to life-threatening bleeding. Early recognition is important because discontinuation of Vancomycin is required for its resolution. The pathophysiology behind VIT is not completely understood. *Ex vivo* assays demonstrate that the antibodies bind platelets only in the presence of Vancomycin [5] as has been described for other drugs such as quinine and quinidine [6]. It has been proposed that Vancomycin acts as a hapten, binding to the platelet's surface glycoprotein IIb and/or IIIa leading to antibody formation [7]. An alternative explanation suggests that the drug binds initially to circulating antibodies (not to the large protein carrier as is classically described for the generation of hapten-specific antibodies), changing the antibody's configuration so that it can more tightly bind the platelet's *α*IIb/*β*3 integrin (GPIIa/IIIb) [8]. Whatever the sequence of initiating events, these antibodies, in the continued presence of Vancomycin, coat platelets leading to complement activation and platelet destruction [9]. Unlike heparin-induced thrombocytopenia which can cause both bleeding and thrombosis, Vancomycin-induced thrombocytopenia only increases the risk of bleeding [10]. One published case series of VIT indicated that one-third of the patients experienced severe bleeding, but none had thrombosis [5]. This is consistent with the described patient who developed a life-threatening intrapulmonary hemorrhage, but without any evidence of venous or arterial thrombosis.

Reports suggest that at least 6 days are needed after an initial Vancomycin exposure to develop VIT with a mean of 8 days to reach platelet nadir after first exposure [5, 7]. However, the interval can be significantly shorter in cases of reexposure to Vancomycin [11–13]. In the described patient, thrombocytopenia started within 3 days of receiving Vancomycin raising the possibility that this was not a primary, but an anamnestic antibody response due to prior treatment with Vancomycin. A review of the patient's chart did not reveal previous treatment with Vancomycin during his current hospitalization, but the presence of both IgM and IgG antiplatelet antibodies suggests he may have previously been exposed to this antibiotic at an outside hospital.

The normalization of the platelet count in VIT is thought to require a decrease in serum Vancomycin levels below the threshold necessary to activate the anti-platelet antibodies [14]. A mean of 7.2 days between drug discontinuation and a platelet count greater than 150 × 10^9^/L (off steroids) has been reported [5]. In contrast, this patient's platelets did not stay above 150 × 10^9^/L off steroids until 18 days after Vancomycin discontinuation. His prolonged duration of thrombocytopenia was likely due to decreased Vancomycin clearance due to the patient's acute kidney injury. Dialysis was paused when his platelets decreased to less than 10 × 10^9^/L due to concerns over potential bleeding; this may have prolonged the patient's thrombocytopenia since it allowed the Vancomycin to remain in the patient's serum longer than had we continued dialysis. Although treatments such as IVIG, plasma exchange, platelets transfusion, and steroid have shown minimal benefits in previously published reports [5], we chose to empirically treat the patient with steroids and IVIG due to his intrapulmonary hemorrhage. His airway bleeding stopped, and his platelets began to increase 48 hours after beginning steroids, at a time when his Vancomycin trough was still 10 micrograms/ml, suggesting that the steroids and/or IVIG may have been helpful.

The assay for Vancomycin-dependent antibodies is a flow cytometry-based detection of drug-dependent platelet antibodies; it is a send-out test done by Versiti Wisconsin Inc. and, at, present, cannot be used to diagnose VIT in the acute setting. Therefore, awareness that Vancomycin can cause thrombocytopenia is the first line of defense for diagnosing this condition.

## References

[1] Vidal C., González Quintela A., Fuente R. (1992). Toxic epidermal necrolysis due to vancomycin. *Annals of Allergy*.

[2] Marraffa J., Guharoy R., Duggan D., Rose F., Nazeer S. (2003). Vancomycin-induced thrombocytopenia: a case proven with rechallenge. *American College of Clinical Pharmacy Journal*.

[3] Kuruppu J. C., le T. P., Tuazon C. U. (1999). Vancomycin-associated thrombocytopenia: case report and review of the literature. *American Journal of Hematology*.

[4] Aster R. H. (2005). Drug-induced immune cytopenias. *Toxicology*.

[5] Von Drygalski A., Curtis B. R., Bougie D. W. (2007). Vancomycin-induced immune thrombocytopenia. *The New England Journal of Medicine*.

[6] Kunicki T., Russell N., Nurden A. T., Aster R. H., Caen J. P. (1981). Further studies of the human platelet receptor for quinine-and quinine-dependent antibodies. *The Journal of Immunology*.

[7] Mohammadi M., Jahangard-Rafsanjani Z., Sarayani A., Hadjibabaei M., Taghizadeh-Ghehi M. (2017). Vancomycin-induced thrombocytopenia: a narrative review. *Drug Safety Journal*.

[8] Bougie D. W., Peterson J., Rasmussen M., Aster R. H. J. B. (2015). Mechanism of quinine-dependent monoclonal antibody binding to platelet glycoprotein IIb/IIIa. *The Journal of the American Society of Hematology*.

[9] Rondina M. T., Walker A., Pendleton R. C. (2010). Drug-induced thrombocytopenia for the hospitalist physician with a focus on heparin-induced thrombocytopenia. *Hospital Practice Journal*.

[10] Zinkovsky D. A., Antonopoulos M. S. (2008). Heparin-induced thrombocytopenia: overview and treatment. *Pharmacy and therapeutics journal*.

[11] Mizon P., Kiefel V., Mannessier L., Mueller‐Eckhardt C., Goudemand J. (1997). Thrombocytopenia induced by vancomycin-dependent platelet antibody. *Vox Sang Journal*.

[12] Ruggero M. A., Abdelghany O., Topal J. E. (2012). Vancomycin-induced thrombocytopenia without isolation of a drug-dependent antibody. *Pharmacotherapy Journal*.

[13] Zenon G. J., Cadle R. M., Hamill R. J. (1991). Vancomycin-induced thrombocytopenia. *Archives of Internal Medicine*.

[14] Curtis B. R., McFarland J. G., Wu G. G., Visentin G. P., Aster R. H. (1994). Antibodies in sulfonamide-induced immune thrombocytopenia recognize calcium-dependent epitopes on the glycoprotein IIb/IIIa complex. *Blood*.

